# Supplementary material for: Development of Apoptotic-Cell-Inspired Antibody–Drug Conjugate for Effective Immune Modulation
Source: Int J Mol Sci. 2023 Nov 7;24(22):16036. doi: 10.3390/ijms242216036 (PMC10671176; doi:10.3390/ijms242216036)
Supplement: Supplementary file 1 [file ijms-24-16036-s001.zip › ijms-2633748-supplementary.pdf]

Supplementary Materials

Development of Apoptotic Cell Inspired Antibody-Drug Conjugate Toward Effective Immune Modulation

Gyeongwoo Lee [1,2], Taishu Iwase [1,2], Shunsuke Matsumoto [1,2], Ahmed Nabil [1,3] and Mitsuhiro Ebara [1,2,4,*]

1 Research Center for Macromolecules and Biomaterials, National Institute for Materials Science (NIMS), 1-1 Namiki, Tsukuba 305-0044, Japan
2 Graduate School of Pure and Applied Sciences, University of Tsukuba, 1-1-1 Tennodai, Tsukuba 305-8577, Japan
3 Biotechnology and Life Sciences Department, Faculty of Postgraduate Studies for Advanced Sciences (PSAS), Beni-Suef University, Beni-Suef 62511, Egypt
4 Graduate School of Advanced Engineering, Tokyo University of Science, 6-3-1 Katsushika-ku, Shinjuku, Tokyo 125-8585, Japan
* Correspondence: ebara.mitsuhiro@nims.go.jp

Table of contents:

Supplementary experimental section

Figure S1 GPC elution curve of p(HEMA-*co*-(*t*-Bu/Boc)MPS) synthesized by (a) free radical polymerization and (b) RAFT polymerization.

Figure S2 ^{1}H NMR spectra of (a) (*t*-Bu/Boc)MPS monomer, (b) p(HEMA-*co*-(*t*-Bu/Boc)MPS), and (c) p(HEMA-*co*-MPS).

Figure S3 Fluorescence intensity of p(HEMA-*co*-(*t*-BuO/Boc)MPS) and p(HEMA-*co*-MPS).

Figure S4 GPC elution curve of goat anti-mouse IgG and IgG-p(HEMA-*co*-MPS) 1:2.

Figure S5 SDS-PAGE analysis of Goat anti-mouse IgG, p(HEMA-*co*-MPS), and IgG-p(HEMA-*co*-MPS) with various polymer feed ratio, before dye is fully washed.

Scheme S1. Schematic diagram of antibody binding affinity measurement via enzyme-linked immunosorbent assay.

Supplementary experimental section

Proton nuclear magnetic resonance measurement

Synthesis of 2-methacryloyloxyethyl phosphorylserine (MPS) monomer, Poly(2-hydroxyethyl methacrylate-*co*-(tertiary butyl/tert-butyloxycarbonyl) 2-methacryloyloxyethyl phosphorylserine) (p(HEMA-*co*-(*t*-Bu/Boc)MPS), and Poly(2-hydroxyethyl methacrylate-*co*-2-methacryloyloxyethyl phosphorylserine) (p(HEMA-*co*-MPS)was characterized using proton nuclear magnetic resonance (^{1}H NMR, JEOL, Tokyo, Japan). All samples were dissolved in DMSO-d_6 at a concentration of 1 mg/mL and measured with 64 scans. Introduction of protective PS group was verified by introduction of tertiary butyl (*t*-Bu) and tert-Butyloxycarbonyl (Boc) peaks at 1.1-1.4 ppm. Polymerization of HEMA and MPS was verified by disappearance of diene peaks in monomers at 5.7 and 6.0, respectively. Deprotection of protective group was verified by disappearance of *t*-Bu and Boc group peaks.

Gel permeation chromatography

To determine molecular weight of p(HEMA-*co*(*t*-Bu/Boc)MPS), mobile phase was prepared by dissolving lithium bromide (LiBr) in dimethylformamide (DMF) at a concentration of 10 mM. Sample was dissolved in mobile phase at a concentration of 1 mg/mL and filtered through 0.45 μm polyvinylidene fluoride syringe filter. Molecular weight was measured by gel permeation chromatography (EcoSEC Elite, TOSOH, Tokyo, Japan). Mobile phase was passed through TSK-GEL® column at a flow rate of 0.800 mL/min. Measured elucidation profile was calculated to molecular weight via comparison with polystyrene standard (Mw: 500-2110000).

The molecular weight of p(HEMA-*co*-MPS) and immunoglobulin g-p(HEMA-*co*-MPS) conjugate were measured in water based GPC (Jasco, Japan). Phosphate buffer saline (PBS) was selected as a mobile phase, and sample was dissolved in PBS at a concentration of 1 mg/mL. All samples were filtered through 0.45 μm cellulose acetate filter and passed through Toso G4000PWXL+guard column at a flow rate of 1 mL/min. Measured elucidation profile was calculated to molecular weight via comparison with polyethylene glycol standard.

Fluorescamine assay

Deprotection of p(HEMA-*co*(*t*-Bu/Boc)MPS) was verified by fluorescamine assay. Fluorescamine reagent was dissolved in DMF at a concentration of 3 mg/mL. Samples were dissolved in dimethyl sulfoxide (DMSO). Samples were moved to each well of 96 well black plate (Corning, USA) as 100 μL, and fluorescamine solution was added to each well as 10 μL. Well was incubated at 25ºC for 30 minutes, and the fluorescence intensity was measured under the excitation at 390 nm and emission at 475 nm.

Sodium dodecyl sulfate polyacrylamide gel electrophoresis (SDS-PAGE)

Molecular weight increase after conjugation was observed by SDS-PAGE. All samples were prepared in PBS at a concentration of 1 mg/mL, and 2-mercaptoethanol. was added diluted 2-fold by Laemmli Sample Buffer Sample was heated at 95ºC for 5 minutes so that the peptide chain can be completely aligned. Polyacrylamide gel (PAG, BIO RAD, USA) was mounted in electrophoresis holder. Tris/Glycine/SDS Buffer poured into electrophoresis holder so that PAG can be immersed completely. 10 μL of sample and molecular weight standard (BIO RAD, USA) was gently moved to PAG gel using pipet. After that, 200 V of voltage was supplied for 30 minutes, and PAG was separated from case and gently moved to Fix buffer (methanol and ethyl acetate in water). It was then stained for 30 minutes by Coomassie Blue staining solution. PAG was washed in wash buffer (methanol and ethyl acetate in water) with shaking, ad wash buffer was replaced until Coomassie blue dye is completely washed.

Flowcytometry

To evaluate macrophage polarization, RAW-Blue macrophage (Sigma, USA) cells were seeded into a 24-well plate at a density of 10^6 cells/well and cultured in Dulbecco's modified Eagle's medium (DMEM; Nacalai, USA) supplemented with 10% fetal bovine serum (FBS; Sigma-Aldrich, USA) and 1% penicillin-streptomycin (Sigma-Aldrich, USA) in a CO_2 incubator. After 24 h, 4 μg/mL of LPS containing PBS was added in 100 μL to each well so that macrophages were polarized to M1 macrophages. After 30 min, each well was aspirated and washed with PBS once, and 1500 μL of DMEM was added again. After that, each sample was added in 300 μL to each well, including PBS, p(HEMA-*co*-MPS) (2.66×10^{-6} M), and IgG-p(HEMA-*co*-MPS) (1.33×10^{-6} M). After 24 h cells were scrapped and collected. Cells were washed with flowcytometry buffer and permeabilized by permeabilization buffer set (Invitrogen, USA) following manufacturer's instructions. Cells were stained with FITC labeled anti-mouse iNOS antibody (BDScience, USA) and measured with Flowcytometry (FACsAria, BDScience, USA).

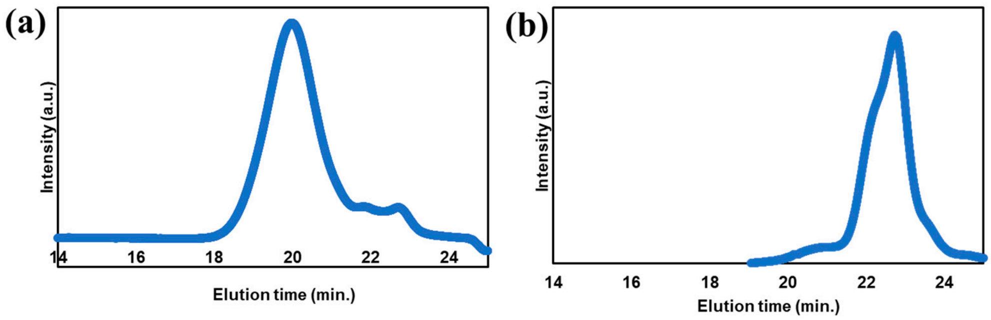

Figure S1 GPC elution curve of p(HEMA-*co*-(*t*-Bu/Boc)MPS) synthesized by (a) free radical polymerization and (b) RAFT polymerization.

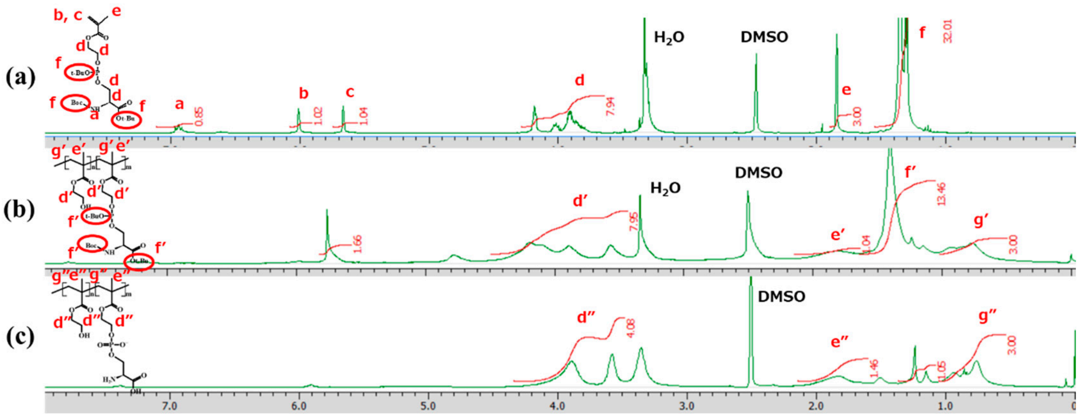

Figure S2 ¹H NMR spectra of (a) (*t*-Bu/Boc)MPS monomer, (b) p(HEMA-*co*-(*t*-Bu/Boc)MPS), and (c) p(HEMA-*co*-MPS).

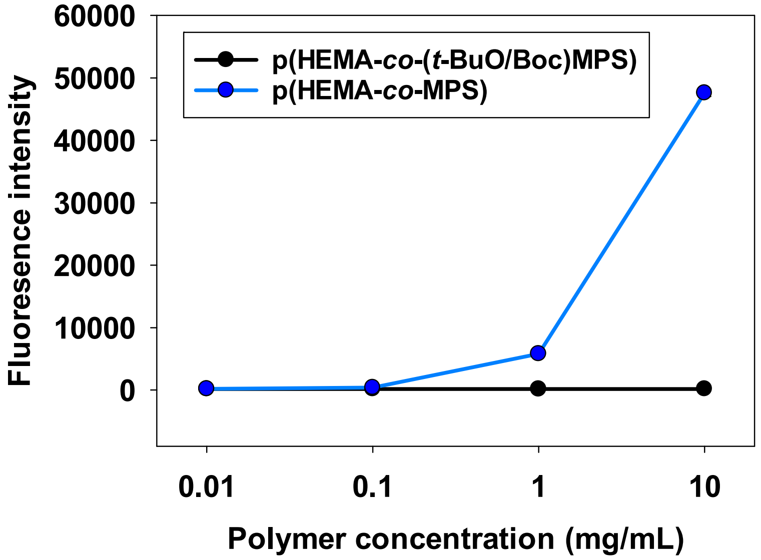

Figure S3 Fluorescence intensity of p(HEMA-*co*-(*t*-BuO/Boc)MPS) and p(HEMA-*co*-MPS).

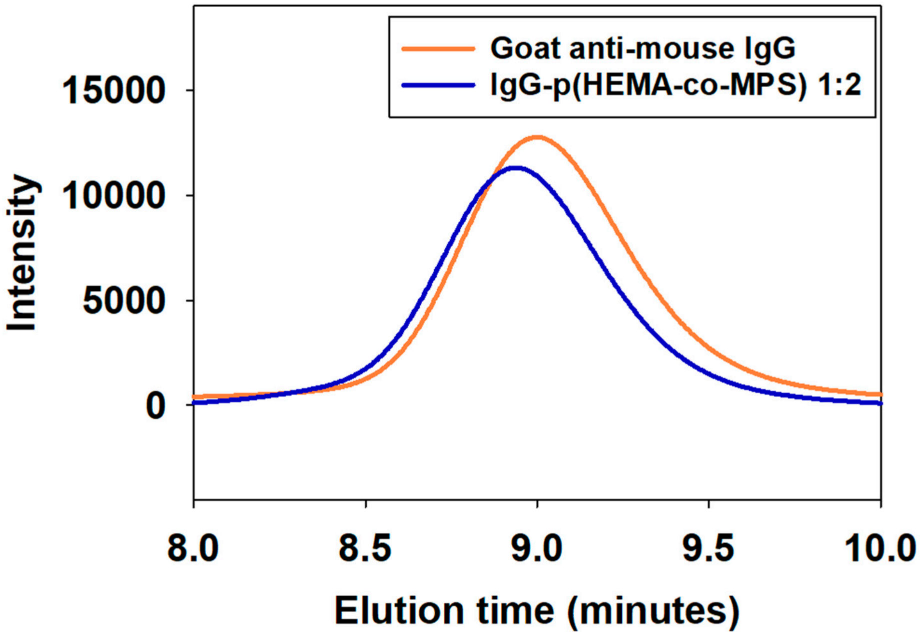

Figure S4 GPC elution curve of goat anti-mouse IgG and IgG-p(HEMA-*co*-MPS) 1:2.

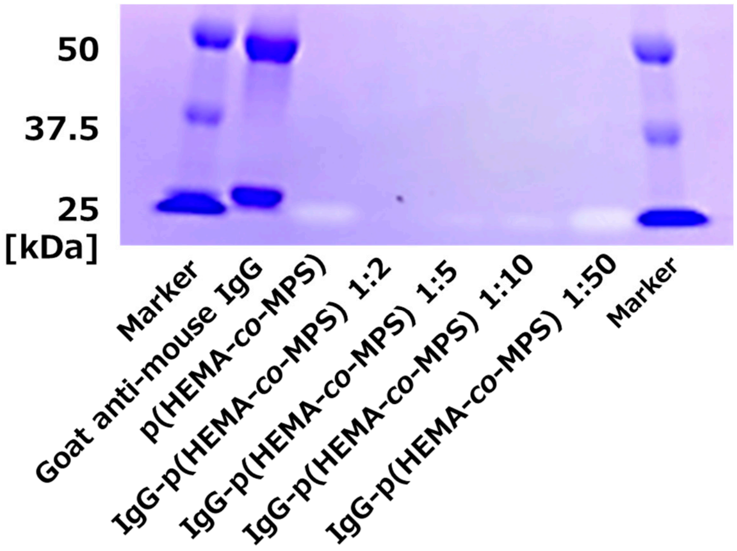

Figure S5 SDS-PAGE analysis of Goat anti-mouse IgG, p(HEMA-*co*-MPS), and IgG-p(HEMA-*co*-MPS) with various polymer feed ratio, before dye is fully washed.

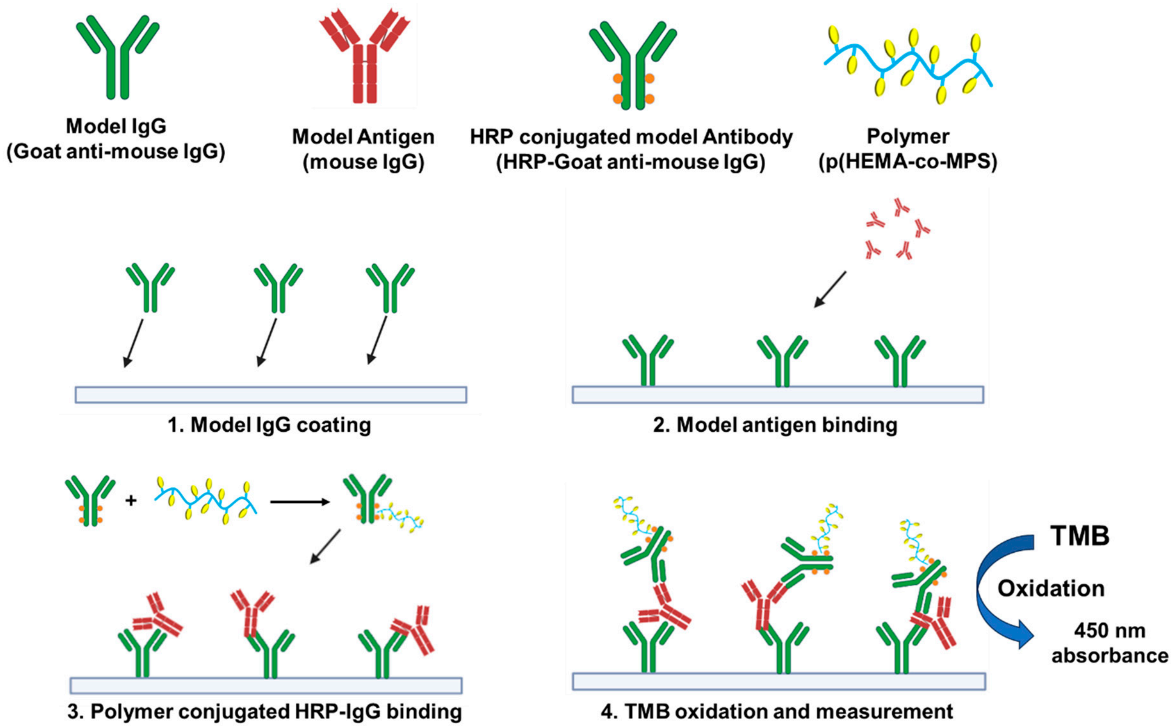

Scheme S1. Schematic diagram of antibody binding affinity measurement via enzyme-linked immunosorbent assay.
